# Bayesian Multiparameter Evidence Synthesis to Inform Decision Making: A Case Study in Metastatic Hormone-Refractory Prostate Cancer

**DOI:** 10.1177/0272989X18788537

**Published:** 2018-08-13

**Authors:** Sze Huey Tan, Keith R. Abrams, Sylwia Bujkiewicz

**Affiliations:** Biostatistics Research Group, Department of Health Sciences, University of Leicester, University Road, Leicester, UK (SHT, KRA, SB); Division of Clinical Trials and Epidemiological Sciences, National Cancer Centre Singapore, Singapore (SHT)

**Keywords:** bivariate meta-analysis, Markov multi-state model, prostate cancer

## Abstract

In health technology assessment, decisions are based on complex cost-effectiveness models that require numerous input parameters. When not all relevant estimates are available, the model may have to be simplified. Multiparameter evidence synthesis combines data from diverse sources of evidence, which results in obtaining estimates required in clinical decision making that otherwise may not be available. We demonstrate how bivariate meta-analysis can be used to predict an unreported estimate of a treatment effect enabling implementation of a multistate Markov model, which otherwise needs to be simplified. To illustrate this, we used an example of cost-effectiveness analysis for docetaxel in combination with prednisolone in metastatic hormone-refractory prostate cancer. Bivariate meta-analysis was used to model jointly available data on treatment effects on overall survival and progression-free survival (PFS) to predict the unreported effect on PFS in a study evaluating docetaxel with prednisolone. The predicted treatment effect on PFS enabled implementation of a 3-state Markov model comprising stable disease, progressive disease, and dead states, while lack of the estimate restricted the model to a 2-state model (with alive and dead states). The 2-state and 3-state models were compared by calculating the incremental cost-effectiveness ratio (which was much lower in the 3-state model: £22,148 per quality-adjusted life year gained compared to £30,026 obtained from the 2-state model) and the expected value of perfect information (which increased with the 3-state model). The 3-state model has the advantage of distinguishing surviving patients who progressed from those who did not progress. Hence, the use of advanced meta-analytic techniques allowed obtaining relevant parameter estimates to populate a model describing disease pathway in more detail while helping to prevent valuable clinical data from being discarded.

In health technology assessment (HTA), reimbursement decisions for new health technologies are made based on cost-effectiveness models. Such often complex models are implemented using estimates of effectiveness, health-related quality of life (HRQoL), and cost. Effectiveness estimates are usually obtained from the systematic literature review and meta-analysis of randomized controlled trials (RCTs), which are designed to give an estimate of the treatment effect on the primary clinical outcome. The choice of the outcome measures for RCTs and reporting of findings rarely take into consideration what is important from the HTA perspective. There is often a lot of heterogeneity in reporting of clinical outcomes due to, for example, variety of scales on which effectiveness can be measured, different time points at which different studies report their outcomes, or different control arms. Relevant outcomes may not be reported due to poor study design, outcome reporting bias, or problems with outcome measurement. Although one would expect the outcome reporting bias (or other mechanisms for not reporting important information) to have decreased over the years as trials are the subject of increased scrutiny, there is still a nonnegligible issue with reporting of the outcomes of RCTs. For example, a systematic review of pharmacological treatments in advanced colorectal cancer by Ciani et al.^1^ found that only 41 of 101 studies reported the treatment effect on progression-free survival (PFS). Among 75 studies published between 2003 and 2010, 36% did not report the treatment effect on PFS, and 54% of 26 studies published between 2011 and 2012 also did not report the treatment effect on this outcome. Another example is a meta-analysis in advanced non–small cell lung cancer (NSCLC) by Créquit et al.^2^ where it was not possible to obtain the treatment effect on PFS in 17 of 102 studies, 9 of them published between 2000 and 2010 and 8 of them published between 2011 and 2016. Such problems with reporting important outcomes may lead to difficulties with populating a cost-effectiveness model with appropriate parameters.

Bayesian statistics provides a flexible framework for modeling complex data structures by allowing multiple parameters to be modeled simultaneously. This is particularly useful when multiple data sources need to be brought together, which can be achieved by the use of multiparameter evidence synthesis. Network meta-analysis (NMA) facilitates simultaneous comparison of multiple treatment options with an aim of obtaining effectiveness estimates for all possible treatment contrasts, including those that may not be directly reported by any RCTs.^3^ Multivariate meta-analysis (MvMA) allows joint modeling of treatment effects on multiple outcomes with the aim of obtaining pooled effects on all the outcomes while taking into account the correlation between them.^4–6^ There are many advantages of MvMA, including 1) potentially increased precision of effectiveness estimates, which can lead to increased precision of other estimates, such as HRQoL^7^; 2) inclusion of all relevant evidence from studies reporting relevant outcomes (other than the main outcome of interest), preventing valuable data from being discarded^7^; and 3) potentially reduced outcome reporting bias.^8^ In this article, we propose the use of bivariate random-effects meta-analysis (BRMA) for the purpose of predicting unreported treatment effects in individual studies, rather than obtaining overall pooled effects,^6,9,10^ aiming to inform a complex HTA modeling framework.

A multistate Markov model is one of the most frequently used decision models in HTA. The number of health states in the model depends on the disease area, and the states should be chosen to represent clinically and economically important events and be mutually exclusive, such as a 3-state model, including asymptomatic state, progressive disease state, and dead state.^11^ To populate a 3-state model in cancer, the transition probabilities between the states are obtained from data on both overall survival (OS) and PFS. When data are not available to estimate all parameters of the model, the model may need to be simplified, which conflicts with its purpose to simulate real-life scenarios.

We illustrate how multiparameter evidence synthesis can be applied to fully utilize all available evidence to inform parameters of a Markov model. To demonstrate this methodology, we apply the methods to inform cost-effectiveness analysis of docetaxel in prostate cancer. In the technology assessment of docetaxel, Collins et al.^12^ constructed a 2-state Markov model consisting of the alive and dead states. Reviewing the evidence on the effectiveness of docetaxel made it apparent that relevant evidence of the treatment effect on PFS was not available for docetaxel at the HTA submission stage, limiting the development of the decision model to the two states only. We demonstrate how the use of MvMA can lead to obtaining relevant estimates necessary to populate a 3-state Markov model, including a progression state.

Modeling the natural course of the disease through the relevant number of states corresponding to the associated utility, cost, and transition probabilities (rather than merely modeling available data) is very important from the point of view of the structural uncertainty of the health economic model.^13^ In other disease areas, Markov models may include a different number of unique states related to the stages specific to the particular disease. Transition probabilities will then be based on multiple effectiveness parameters related to those states, which can be modeled jointly through the multivariate meta-analysis, taking into account of missing data^6^ likely present when several outcomes are involved.

We present the use of the methodology for one specific case study of prostate cancer, but we believe that its applicability can be much broader, extending to a range of different disease areas, potentially other than cancer or to predict treatment effect on outcomes other than PFS. For example, in early assessment of new therapies, treatment effect on PFS may be recorded but more data may need to be collected before the treatment effect on OS can be measured. Bivariate meta-analysis can be used to predict unmeasured treatment effect on OS to inform a cost-effectiveness model in a similar manner as demonstrated in our case study in this article.

## Methods

### Motivating Example and Sources of Evidence

In 2007, the National Institute for Health and Care Excellence (NICE) carried out a technology appraisal of docetaxel in combination with either prednisone or prednisolone (D + P) as treatments for metastatic hormone-refractory prostate cancer (mHRPC).^12^ The technology appraisal aimed to evaluate the clinical and cost-effectiveness of the combination therapy. The evidence base for the meta-analysis in this HTA included 4 studies that investigated interventions that were licensed at the time of the HTA submission: D + P, mitoxantrone plus prednisone (M + P), prednisolone alone (P), mitoxantrone plus hydrocortisone (M + H), and hydrocortisone alone (H). Data from the 4 RCTs,^14–17^ listed in Table 1, are included in our example and referred to as the HTA set. None of the studies reported the effect of docetaxel on PFS, required to populate a 3-state Markov model, and only 1 trial reported the effect of docetaxel on OS (TAX 327^16^). The details of the systematic review conducted by Collins et al^12^ are included in Supplemental Appendix A, which also includes a set of studies of unlicensed treatments used to inform some of the model parameters in this article.

**Table 1:** Randomized Controlled Trials Used as an Evidence Base for the Clinical Effectiveness Analysis in the HTA Report.

Trial	Year	No. of Arms	Reference Treatment	Comparative Treatment(s)	Total No. of Patients	OS Data	PFS Data
CCI-NOV22^17^	1996	2	M + P	P	161	Yes	Yes
CALGB 9182^15^	1999	2	M + H	H	242	Yes	Yes
Berry et al.^14^	2002	2	M + P	P	120	Yes	Yes
TAX 327^16^	2004	3	M + P	D + P	1006	Yes	No
				D1+ P			

D + P, 3-weekly docetaxel plus prednisone or prednisolone; D1+P, weekly docetaxel plus prednisone; H, hydrocortisone alone; HTA, health technology assessment; M + H, mitoxantrone plus hydrocortisone; M + P, mitoxantrone plus prednisone or prednisolone; OS, overall survival; P, prednisolone or prednisolone alone; PFS, progression-free survival.

### Methods of Evidence Synthesis

**Figure 1:**
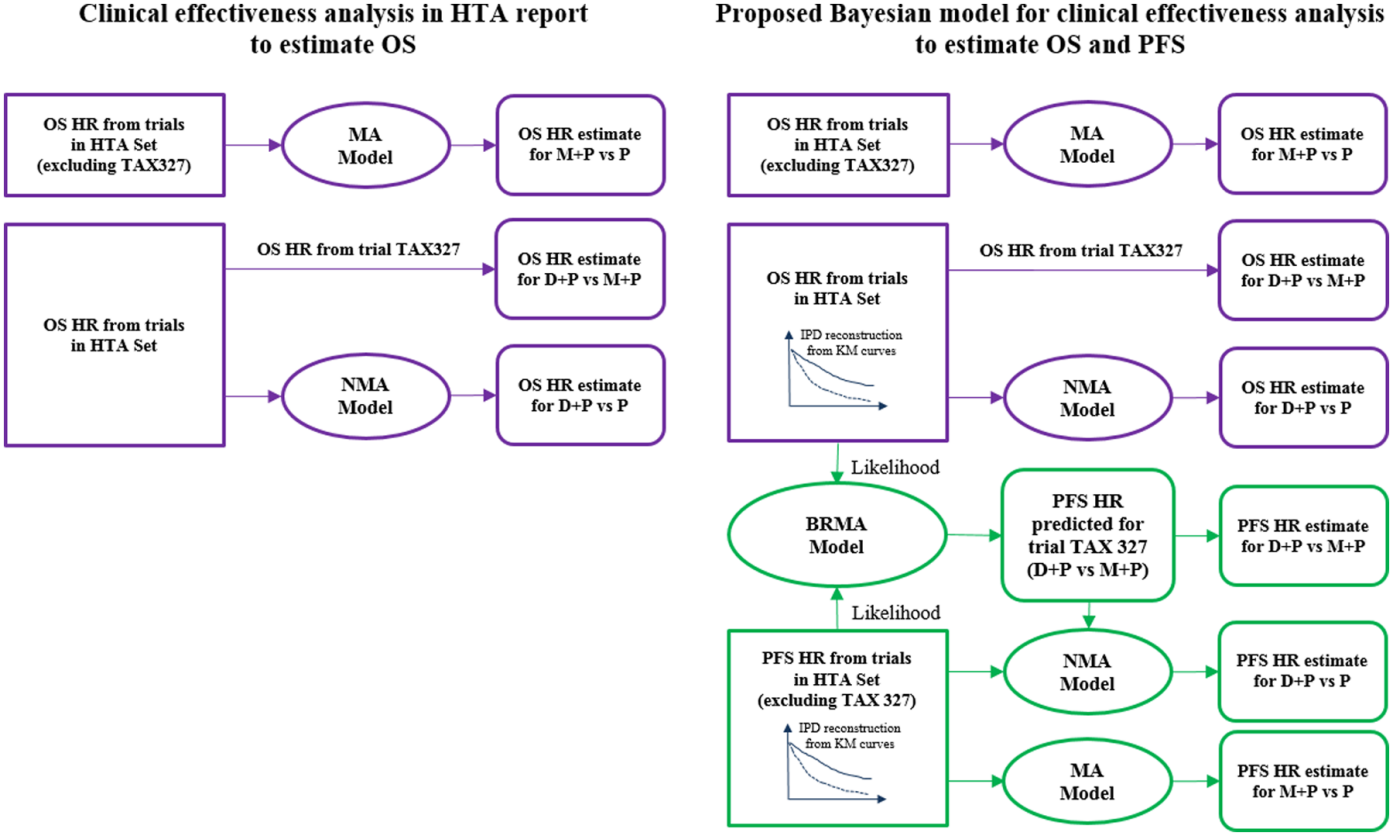
Diagram for the clinical effectiveness analysis. Left-hand side represents the evidence used in the health technology assessment (HTA) report by Collins et al.^12^ and the WinBUGS 2-state model, while the right-hand side represents the evidence used in the WinBUGS 3-state model. The rectangles with straight corners represent the data, ellipses represent the meta-analytic methods (MA, NMA, and BRMA), and the rectangles with rounded corners show resulting pooled effects for different treatment contrasts. Purple color is used to denote estimates and methods for OS (top part of the diagram) and green for the PFS (bottom part). BRMA, bivariate random-effects meta-analysis; D + P, docetaxel plus prednisone or prednisolone; HR, hazard ratio; HTA, health technology assessment; M + P, mitoxantrone plus prednisone or prednisolone; MA, meta-analysis; NMA, network meta-analysis; OS, overall survival; P, prednisolone or prednisolone alone; PFS, progression-free survival.

Figure 1 shows schematically the evidence base and the procedures of obtaining the estimates of effectiveness for all relevant treatment comparisons for the HTA 2-state model and for the reproduced 2-state model on the left-hand side (LHS) as well as the 3-state model on the right-hand side (RHS) of the diagram. Collins et al.^12^ obtained the estimates of the hazard ratios (HRs) for OS for the HTA (LHS diagram) in the following way. The authors conducted a meta-analysis combining 3 trials of mitoxantrone with a corticosteroid (P or H) v. corticosteroid alone by grouping the corticosteroids P and H. We denote them here as P and the resulting HR as M + P v. P. The HR for the treatment effect of D + P v. M+P was obtained directly from the TAX 327 trial. The NMA model of all 4 trials was used to obtain the HR comparing D + P v. P. No evidence on PFS was used in the HTA model.

In our analysis, represented in the RHS diagram of Figure 1, we used similar methods to obtain the effectiveness estimates for OS (top of the graph) as in the HTA report (HR for M + P v. P from meta-analysis of the 3 trials of M + P v. P, HR for D + P v. M + P from trial TAX 327 and for D + P v. P using NMA). However, to unify the scale across all studies for both OS and PFS and to be able to use comparable estimates between 2-state and 3-state models, where possible, we used the HRs obtained from the reconstructed individual patient data (IPD) from Kaplan-Meier curves. To obtain the HR for PFS for M + P v. P, we also used meta-analysis of the same 3 trials as for OS but this time combining the estimates of the treatment effect on PFS. Unlike for OS, the effect of D + P v. M + P on PFS was not available in trial TAX 327. This estimate was vital to populate the 3-state model. To obtain it, the bivariate meta-analysis of data from the 4 trials in the HTA set was used by modeling jointly log HRs on PFS and OS, with HR on PFS for TAX 327 coded as a missing effect, which was then predicted from the model. For uniformity across the treatment effects on OS and PFS, we used the reconstructed IPD on both outcomes in the BRMA. The predicted HR on PFS for trial TAX 327 was then used in the NMA (similarly as for the OS) to obtain the estimate of the effect of D + P v. P on PFS.

Methods for obtaining the summary trial data for this analysis on an appropriate scale are described in the Data extraction and reconstruction section. The meta-analytic method for combining the evidence on PFS and OS for the purpose of predictions is described in the Bivariate meta-analysis section, while the Network meta-analysis section discusses briefly NMA.

#### Data extraction and reconstruction

For the purpose of evidence synthesis, summary data on effectiveness measured on OS and PFS were analyzed on the log HR scale, to allow for assumption of normality of the treatment effect estimates. To obtain the estimates on this scale, IPD on OS and PFS for each of the RCTs were reconstructed from their Kaplan-Meier survival curves, if reported, using the method by Guyot et al.^18^ Reconstructed IPD allow log HRs and corresponding standard errors (SEs) to be estimated using survival analysis instead of crude estimation using median survival times and log-rank test *P*-values reported in the RCTs. Survival analyses, using the Cox model, were performed on the reconstructed IPD of the 4 RCTs in the HTA set for OS and 2 RCTs in the HTA set for PFS (only CALGB 9182^15^ and Berry et al.^14^ reported Kaplan-Meier survival curves for PFS) to estimate the log HRs for the meta-analysis. The estimate of log HR on PFS for trial CCI-NOV22^16^ was obtained from the HTA report^12^ as it was not reported in the trial paper. Trial TAX 327 did not report the PFS endpoint.

#### Bivariate meta-analysis

Bivariate random-effects meta-analysis was used for the purpose of predicting the treatment effect on PFS in the docetaxel trial (TAX 327) by modeling treatment effects measured by log HRs on OS and PFS jointly. In this model, $Y_{\mathrm{OS}}$ and *Y_PFS_*, the treatment effects on OS and PFS on the log HR scale, are assumed to be normally distributed and correlated:

(1) $$\left(\begin{array}{c} Y_{\mathrm{OS},i}\\ Y_{\mathrm{PFS},i} \end{array}\right)\sim \mathrm{Normal}\left(\left(\begin{array}{c} \mu_{\mathrm{OS},i}\\ \mu_{\mathrm{PFS},i} \end{array}\right),\Sigma_{i}\right),$$

with the within-study variance-covariance matrices $\Sigma_{i}=\left(\begin{array}{cc} \sigma_{\mathrm{OS},i}^{2} & \sigma_{\mathrm{OS},i}\sigma_{\mathrm{PFS},i}\rho_{w,i}\\ \sigma_{\mathrm{PFS},i}\sigma_{\mathrm{OS},i}\rho_{w,i} & \sigma_{\mathrm{PFS},i}^{2} \end{array}\right)$ comprising the within-study standard errors of the estimates, $\sigma_{\mathrm{OS},i}$ and $\sigma_{\mathrm{PFS},i}$, and the within-study correlations $\rho_{w,i}$ between the estimates in each study i. The treatment effects $Y_{\mathrm{OS},i}$ and $Y_{\mathrm{PFS},i}$ are the estimates the true effects, $\mu_{\mathrm{OS},i}$ and $\mu_{\mathrm{PFS},i}$, which are also correlated and can be modeled in the form of the product of univariate conditional normal distributions, the product normal formulation^6,9,10^:

(2) $$\begin{array}{cc} & \mu_{\mathrm{OS},i}\sim \mathrm{Normal}\left(\eta_{\mathrm{OS}},\psi_{\mathrm{OS}}^{2}\right)\\ & \mu_{\mathrm{PFS},i}|\mu_{\mathrm{OS},i}\sim \mathrm{Normal}\left(\eta_{\mathrm{PFS},i},\psi_{\mathrm{PFS}}^{2}\right)\\ & \eta_{\mathrm{PFS},i}=\lambda_{0}+\lambda_{1}\left(\mu_{\mathrm{OS},i}-\bar{\mu_{\mathrm{OS},i}}\right) \end{array}$$

where $\psi_{\mathrm{OS}}^{2}$ is equal to the between-studies variance $\tau_{\mathrm{OS}}^{2}$ (heterogeneity parameter) of the treatment effects on OS, and $\psi_{\mathrm{PFS}}^{2}$ is the conditional between-studies variance of the treatment effect on PFS conditional on the treatment effect on OS, which is related to the heterogeneity parameter $\tau_{\mathrm{PFS}}^{2}$; $\psi_{\mathrm{PFS}}^{2}=\tau_{\mathrm{PFS}}^{2}-\lambda_{1}^{2}\tau_{\mathrm{OS}}^{2}.$ The slope $\lambda_{1}=\rho_{b}\tau_{\mathrm{PFS}}/\tau_{\mathrm{OS}}$ and $\rho_{b}$ is the between-studies correlation. Prior distributions are placed on the between-studies correlation and variances: for example, $\rho_{b}\sim \mathrm{Uniform}\left(-1,1\right)$ and half normal distributions for the standard deviations, $\tau_{\mathrm{OS}}\sim \mathrm{HNormal}\left(0,10^{3}\right)$ and $\tau_{\mathrm{PFS}}\sim \mathrm{HNormal}\left(0,10^{3}\right)$, which give implied prior distributions on $\lambda_{1}$, $\psi_{\mathrm{OS}}$ and $\psi_{\mathrm{PFS}}$ obtained using the above relationships between the parameters. Prior distributions are also placed on the intercept $\lambda_{0}\sim \mathrm{Normal}\left(0,10^{3}\right)$ and the within-study correlations $\rho_{w,i}\sim \mathrm{Uniform}\left(-1,1\right)$. The pooled treatment effects are $HR_{\mathrm{OS}}=\exp(\eta_{\mathrm{OS}})$ and $HR_{\mathrm{PFS}}=\exp\left(\lambda_{0}\right)$. Further assumption about the exchangeability of population variances was made, as in Bujkiewicz et al.,^6^ to predict the standard error corresponding to the missing log HR on PFS, comparing D + P v. M + P, in TAX 327.

The unreported effect on PFS in trial TAX 327, $Y_{\mathrm{PFS},\mathrm{TAX}\;327},$is predicted directly from the Markov chain Monte Carlo (MCMC) simulation. In the ordinary approach to MvMA, predicted effects (on outcomes that are not reported) are by-products of the analysis that contribute to the pooled effects.^6^ Here we exploit this by using the predicted value directly to inform the decision model.

#### Network meta-analysis

NMA allows for the comparisons of interventions when there is no head-to-head RCT that compared them directly by evaluating the difference between the interventions through at least 1 common comparator.^19,20^ It combines both direct and indirect evidence from multiple studies of multiple interventions.^3^ NMA was used in this study to obtain HRs for both OS and PFS comparing D + P v. P, which is an alternative contrast that can be used in a health economic model, as done by Collins et al.^12^ for OS in the 2-state model.

### Methods of Cost-effectiveness Analysis

**Figure 2:**
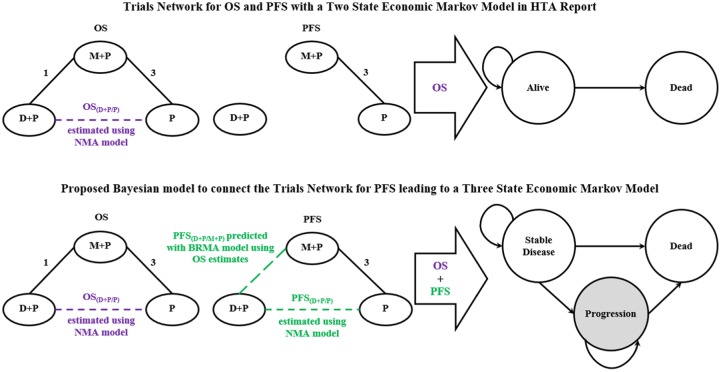
Original HTA model (top) and proposed Bayesian evidence synthesis with BRMA to predict PFS for the specification of a 3-state economic Markov model (bottom). BRMA, bivariate random-effects meta-analysis; D + P, docetaxel plus prednisone or prednisolone; HTA, health technology assessment; M + P, mitoxantrone plus prednisone or prednisolone; NMA, network meta-analysis; OS, overall survival; P, prednisolone or prednisolone alone; PFS, progression-free survival.

Figure 2 shows schematically the evidence base in the form of network diagrams for both OS and PFS on the LHS and the structure of the Markov model on the RHS. The top part of the figure corresponds to the HTA model by Collins et al.^12^ The bottom part summarizes the analysis developed in this article. Collins et al.^12^ specified a 2-state Markov model, including the alive and dead states, which assessed the cost-effectiveness of D + P for the treatment of mHRPC. To model the natural pathway of the disease in more detail, we used the predicted estimate for the treatment effect of D + P on PFS to populate the 3-state model, which distinguished the alive patients with the disease between those who are stable and those who progressed by assigning them 2 separate states: stable disease (StD) and progressive disease (PD) states, which together with the dead state amount to the 3-state model.

For the comparison of the results of the proposed 3-state model with the results of the 2-state model reported in the HTA, the 2-state model was reproduced to ensure that the models (2-state and 3-state) are comparable in terms of the software used (Excel software was used to implement the original HTA model). For clarity, the reproduced 2-state model will be called the “WinBUGS 2-state model,” and the original 2-state model in the HTA report will be called the “HTA 2-state model” in this article. Similar to the HTA 2-state model, both the WinBUGS 2-state model and WinBUGS 3-state model were run for 180 cycles, where 1 cycle represented 1 month and the time horizon was 15 years. A cohort size of 10,000 was used in each of the models. When constructing the 3-state model, where possible, we used the same parameters as in the HTA 2-state model. In the remainder of this section, we highlight the methods most relevant for the extension of the 2-state model to the 3-state model and report the full set of modeling techniques in the supplementary materials for completeness.

#### Transition probabilities

Similar to the HTA 2-state model, the transition probabilities for the WinBUGS 2-state model were estimated using the Weibull parameters (reported in the HTA report) obtained from the trial TAX 327 (using M + P as the baseline treatment). For the 3-state model, which incorporated a PD state, the transition probabilities for transition from the StD state to the PD state needed to be estimated using data on PFS, which the trial TAX 327 did not report. We estimated these transition probabilities for M + P by applying the Weibull survival model to reconstructed PFS IPD from a Southwest Oncology Group (SWOG) trial.^21^ The SWOG trial was one of the RCTs of unlicensed drugs used in the sensitivity analysis in the HTA report and was the most comparable trial with TAX 327. This was particularly apparent when examining the Kaplan-Meier curves for OS (corresponding to the baseline treatment M + P, which the 2 trials had in common), obtained from the reconstructed IPD and shown in Supplemental Figure SC1. The Kaplan-Meier curve for the SWOG trial was comparable to the one obtained for the TAX 327 trial. Further details about the studies are included in Supplemental Appendix A and the justification of the choice of the SWOG trial in Supplemental Appendix C.2. The transition probability for D + P was in turn calculated by applying the predicted PFS HR of D + P v. M + P to the transition probability of M + P. The transition probability for transition from the StD state to the dead state (for deaths due to causes other than prostate cancer) was obtained from the cost-effectiveness analysis for advanced hormone-dependent prostate cancer by Lu et al.^22^ and was applied to the model with no uncertainty as 0.005. The parametric survival (Weibull) model was used to implement time dependency of the transition probabilities in the economic models. Details of this analysis are included in Supplemental Appendix B.1.1.

Although IPD were reconstructed for both PFS and OS for the trial selected for estimating the transition probability from the StD state to PD state, the IPD were not paired by patient. Therefore, it would not be possible to estimate the transition probabilities from the PD state to the dead state using parametric survival analysis performed using reconstructed IPD as was the case for the transition probability for the StD state to the PD state (which only required data on PFS). To overcome this issue, the transition probabilities were estimated by assuming that the mean total survival time was equal to the weighted sum of combined survival time from stable disease to progression and then to death and the survival time from stable disease to death when death occurred from other causes. The method is described in more detail in Supplement Appendix B.1.2.

Once the transition probabilities were obtained for the baseline treatment M + P, the HRs (obtained using methods described earlier) were applied to them to obtain the transition probabilities for the other 2 interventions (D + P and P alone). For the 2-state model, HR for OS was used to obtain the transition probabilities from alive to dead states. For the 3-state model, HRs for PFS were used to obtain the transition probabilities from the StD to the PD state.

#### Costs

Cost data comprise the drug acquisition and the administration cost for each intervention, cost of the management of adverse events, and the subsequent follow-up cost that included cost of further chemotherapy after disease progression, management of side effects, and palliative care. Costs for each intervention used in the WinBUGS 2-state and 3-state models were extracted from the HTA report. In the report, costs were categorized into 3 components: 1) the drug acquisition and administration cost, 2) the follow-up cost, and 3) the terminal care cost. In the 3-state model, the follow-up costs were divided into 2 portions corresponding to the StD state and PD state by taking into account that costs of subsequent chemotherapy and hospitalizations accounted for between 70% and 80% of follow-up costs, which most likely occurred after progression, and the remaining follow-up costs (20% to 30%) were related to side effects likely to occur prior to progression (but may also be associated with the subsequent chemotherapy after progression). An annual discount rate of 3.5% was applied from cycle 13 onward, as in the HTA report by Collins et al.^12^ Details of the cost analysis are included in Supplemental Appendix B.2.

#### Quality-adjusted life years

Quality-adjusted life years (QALYs) were used as a measure of effect in the cost-effectiveness analysis. To estimate the QALYs, utility data in the form of HRQoL were required to quantify the health status of patients with mHRPC, as well as the impact the interventions had on the HRQoL (in terms of disease progression and serious adverse events). Quality-of-life data used in the HTA 2-state model by Collins et al.^12^ were extracted from a study conducted by Sandblom et al.^23^ The study was appropriate as it reported HRQoL values using a generic HRQoL instrument, the EuroQoL 5-dimensional (EQ-5D) questionnaire, which is required in submissions for technology assessment by NICE; it used the population representative of the target population of the HTA and provided end-of-life HRQoL values of patients with prostate cancer in their last year before death. Sandblom et al. reported an EQ-5D score of 0.538 ± 0.077 for patients who died of prostate cancer, which was an average value recorded during the last 12 months of a patient’s life. This value was used in the HTA 2-state model as a utility in the alive state. We used the same value in our WinBUGS 2-state model. Since the data were collected from the individuals who were diagnosed at least 9 months earlier and considering that the median survival time is 18 months and the median PFS time is 6 months, these patients (who then died of prostate cancer) were most likely in progressive disease. Consequently, when constructing a 3-state model that distinguished between alive patients in stable disease from those who progressed, we considered this utility value appropriate for the PD state ($U_{\mathrm{PD}}=0.538$) and expected the utility corresponding to the StD state to be higher. Sandblom et al.^23^ also reported EQ-5D values for patients who were still alive after a 12-month follow-up, which was $U_{\mathrm{Surviving}}=0.770\pm 0.015$, and for those who died of other causes: $U_{\mathrm{Other}\;\mathrm{causes}}=0.564\pm 0.067$. Considering that in each cycle, patients in the StD state stay in that state, progress, or die of other causes, we assigned to this state the utility corresponding to the average of the above 3 utilities reported by Sandblom et al.^23^ weighed by the transition probabilities:

(2) $$U_{\mathrm{StD}}=TP_{\mathrm{StD}}U_{\mathrm{Surviving}}+TP_{\mathrm{StDtoPD}}U_{\mathrm{PD}}+TP_{\mathrm{StDtoDead}}U_{\mathrm{Other}\;\mathrm{causes}}.$$

For further details, see Supplemental Appendix B.3. Similar to cost, an annual discount rate of 3.5% was used for discounting the utilities after the first year.

#### Cost-effectiveness analysis

For the assessment of the cost-effectiveness of the interventions in each model, the mean costs and mean QALYs gained for the interventions and the incremental cost-effectiveness ratios (ICERs) for the comparison of the 2 interventions of interest (M + P and D + P) were estimated. Cost effectiveness acceptability curves (CEACs) were generated to compare the 3 interventions. The CEAC and the cost-effectiveness plane were used to compare the proposed 3-state model with the WinBUGS 2-state model (expected to be comparable to the HTA 2-state model) when evaluating the difference between D + P and M + P. To assess the value of further research, population expected value of perfect information (EVPI) was calculated for both the 2-state and the 3-state model.^24^ Population EVPI was estimated from WinBUGS output^25^ assuming a time horizon of the drugs of 12 years, an annual incidence of 9000 patients,^26^ and a discount rate of 3.5%.^27^

### Software Implementation

IPD were reconstructed from the Kaplan-Meier curves using the DigitizeIt^28^ and R^29^ software. Survival analyses were conducted using Stata.^30^ BRMA and the cost-effectiveness models were implemented using MCMC simulations in WinBUGS,^31,32^ with 30,000 MCMC iterations and 15,000 burn-ins (iterations that were discarded) for BRMA and 50,000 iterations and 30,000 burn-ins for the cost-effectiveness models. Output data were processed using R.^29^

## Results

### Clinical Effectiveness

Kaplan-Meier curves for OS were reported for the 4 trials in the HTA set. PFS was not reported for TAX 327, and PFS Kaplan-Meier curve was not reported for CCI-NOV22. Hazard ratios calculated using the reconstructed IPD for individual trials were comparable with the results reported in the trials’ publications. HRs on OS and PFS reported in the original articles and those obtained from the survival analyses of reconstructed IPD are presented in Supplemental Appendix C (Suppl. Tables SC1 and SC2).

Table 2 presents all pooled HRs for OS and PFS reported in the HTA report and those obtained by synthesizing HRs from the reconstructed IPD. Summary estimates for the HR of OS comparing M + P with P obtained using fixed-effect and random-effects meta-analysis were 0.903 (95% credible interval [CrI], 0.751–1.084) and 0.901 (95% CrI, 0.405–2.023) respectively. The estimates differed slightly from those in the HTA report, which were 0.99 (95% confidence interval [CI], 0.82–1.20) for both fixed-effect and random-effects. The difference in the pooled HRs was largely due to the lower HRs obtained using the reconstructed IPD for trials CCI-NOV22^17^, CALGB 9182^15^, and Berry et al.,^14^ compared to the HRs reported in the HTA report. However, the 95% CrI estimated using fixed-effect meta-analysis was comparable to the 95% CI reported in the HTA report. Hazard ratios comparing the effect of D + P v. P on OS were 0.688 (95% CrI, 0.523–0.907) and 0.688 (95% CrI, 0.300–1.604) using fixed-effect and random-effects NMA, respectively, while the HR from random-effects NMA published in the HTA report was 0.75 (95% CI, 0.57–0.99). Similarly, as for the comparison of M + P v. P, the estimates of HRs obtained from the reconstructed IPD were lower than those reported in the HTA report.

**Table 2:** Overall Survival and Progression-Free Survival HRs Estimated from Traditional and Network Meta-Analysis Using Reconstructed IPD.

	HR (95% CI/CrI)		
	Overall Survival		Progression-Free Survival
Evidence Synthesis	Reported in HTA Report	Estimated Using Reconstructed IPD	Estimated Using Reconstructed IPD
Meta-analysis (M + P/P)			
Fixed-effect analysis	0.99 (0.82–1.20)	0.903 (0.751–1.084)	0.641 (0.532–0.772)
Random-effects analysis	0.99 (0.82–1.20)	0.901 (0.405–2.023)	0.619 (0.170–2.048)
Relative estimate (D + P/M + P)	0.76 (0.62–0.94)^a^	0.76 (0.620–0.936)^a^	0.619 (0.393–0.924)^b^
NMA (D + P/P)			
Fixed-effect analysis	Not performed	0.688 (0.523–0.907)	0.397 (0.318–0.496)
Random-effects analysis	0.75 (0.57–0.99)	0.688 (0.300–1.604)	0.383 (0.108–1.290)

CI, confidence interval; CrI, credible interval; D + P, docetaxel plus prednisone or prednisolone; HR, hazard ratio; HTA, health technology assessment; IPD, individual patient data; M + P, mitoxantrone plus prednisone or prednisolone; NMA, network meta-analysis; P, prednisolone or prednisolone alone.

a HRs estimated using TAX 327 trial IPD/reconstructed IPD.

b HR predicted using bivariate random-effects meta-analysis model.

HRs for PFS comparing M + P v. P, obtained from fixed-effect and random-effects meta-analyses of estimates from the reconstructed IPD, were 0.641 (95% CrI, 0.532–0.772) and 0.619 (95% CrI, 0.170–2.048) respectively. No summary estimate for this comparison was reported in the HTA report. The PFS HR for the comparison of D + P v. M + P (for TAX 327), predicted using BRMA, was 0.619 (95% CrI, 0.393–0.942). The HRs comparing the effect of D + P v. P on PFS were 0.397 (95% CrI, 0.318–0.496) and 0.383 (95% CrI, 0.108–1.290) from fixed-effect and random-effects NMA respectively.

### Cost-effectiveness

The net benefit for each intervention, the probability that each intervention was cost-effective, and the population EVPI at £20,000 and £30,000 are presented in Table 3. In terms of the net benefit and the probability of each treatment being cost-effective, there was considerable uncertainty, with the highest probability only being 0.52 for D + P in the 3-state model compared to 0.44 in the 2-state model. Estimated population EVPI was particularly high; at both £20,000 and £30,000, it was above £139 million for both the 2-state and the 3-state model but was higher for the 3-state model, further indicating a high level of uncertainty in the decision problem.

**Table 3:** Probability of Cost-effectiveness, Net Benefit, Population EVPI, Mean Cost, and Mean QALYs.

	Probability of Cost-effectiveness		Net Benefit (£) (95% CrI)		Population EVPI		Mean Cost (£) (95% CrI)	Mean QALYs (95% CrI)
Intervention	£20,000	£30,000	£20,000	£30,000	£20,000	£30,000		
HTA 2-state model								
P	0.39	0.33	NR	NR			NR	NR
M + P	0.39	0.29	NR	NR	NR	NR	NR	NR
D + P (3 weekly)	0.22	0.38	NR	NR			NR	NR
WinBUGS 2-state model								
P	0.32	0.28	4417 (–5119 to 12,360)	12,512 (1648 to 22,500)			11,772 (6127 to 20,280)	0.809 (0.5590 to 1.0760)
M + P	0.36	0.28	5023 (–2327 to 11,680)	13,153 (4116 to 21,800)	139,360,802	203,240,155	11,237 (6855 to 17,030)	0.813 (0.5718 to 1.0580)
D + P (3 weekly)	0.32	0.44	3479 (–5749 to 12,560)	13,149 (1859 to 24,430)			15,862 (9066 to 23,020)	0.967 (0.6746 to 1.2690)
WinBUGS 3-state model								
P	0.31	0.27	6829 (–4364 to 19,720)	15,320 (830 to 33,820)			10,152 (5160.0 to 17,760.0)	0.849 (0.4389 to 1.4400)
M + P	0.34	0.21	7810 (1724 to 13,090)	16,704 (9635 to 23,240)	238,654,042	226,692,356	9977 (5995.0 to 15,250.0)	0.889 (0.7200 to 1.0600)
D + P (3 weekly)	0.35	0.52	7291 (–728 to 15,080)	18,600 (9543 to 27,540)			15,327 (8589.0 to 22,420.0)	1.131 (0.9391 to 1.3260)

CrI, credible interval; D + P, docetaxel plus prednisone or prednisolone; EVPI, expected value of perfect information; HTA, health technology assessment; M + P, mitoxantrone plus prednisone or prednisolone; NR, not reported; P, prednisolone alone; QALY, quality-adjusted life year.

**Figure 3:**
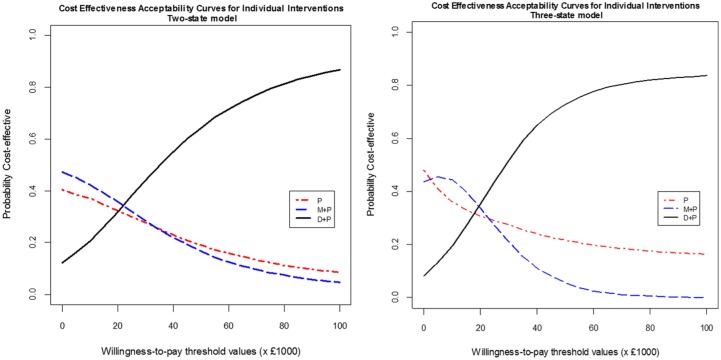
Cost-effectiveness acceptability curves for all 3 interventions: 2-state (left) and 3-state (right) model using direct hazard ratio of P v. M + P. D + P, docetaxel plus prednisone or prednisolone; M + P, mitoxantrone plus prednisone or prednisolone; P, prednisolone or prednisolone alone.

Figure 3 shows CEACs, over a range of willingness-to-pay thresholds, comparing all the interventions using the 2-state and the 3-state model. For the willingness-to-pay thresholds above £20,000, D + P had the highest probability of being cost-effective among all 3 interventions in both models. Fully incremental analysis of cost and QALY in Table 3 shows that P is dominated by M + P in both the 2-state and the 3-state model.

**Figure 4:**
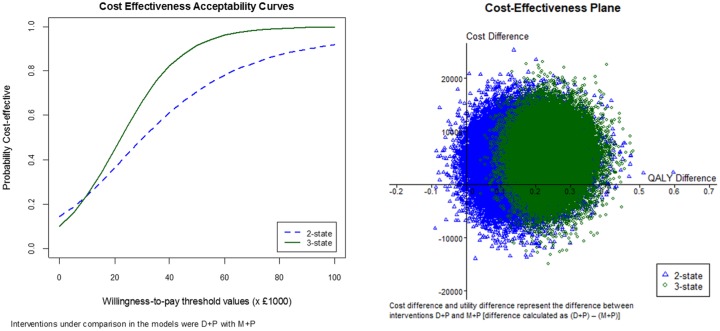
Cost-effectiveness acceptability curves (left) and cost-effectiveness plane (right) for WinBUGS 2-state and 3-state economic models. D + P, prednisone or prednisolone; M + P, mitoxantrone plus prednisone.

Table 4 and Figure 4 show results of the incremental cost-effectiveness analysis. Table 4 presents the differences in mean cost and QALYs along with the ICERs for D + P compared to M + P for all 3 models. Details of the cost of interventions and the mean QALY per patient for each of the interventions in the economic models are presented in Supplemental Appendix C.3. Results showed that the ICER obtained from the proposed 3-state model, using a predicted PFS HR of 0.619 (0.393–0.924) for D + P v. M + P, was £22,148 compared to £30,026 obtained from the WinBUGS 2-state model (£32,706 in the HTA report). Hence, by implementing the 3-state model and taking into account the cost and QALYs in the PD state, the estimated ICER was considerably lower than that of the HTA (or WinBUGS) 2-state model.

**Table 4:** Cost-effectiveness of the HTA 2-State Model and Proposed 3-State Model.

	2-State Model		3-State Model
	HTA	WinBUGS	WinBUGS
Difference in cost, mean (SE)	£5049	£4624 (4407.83)	£5349 (4243.53)
Difference in QALY, mean (SE)	0.15437	0.154 (0.0676)	0.242 (0.0526)
ICER	£32,706	£30,026	£22,148

HTA, health technology assessment; ICER, incremental cost-effectiveness ratio; QALY, quality-adjusted life year; SE, standard error.

Figure 4 shows CEACs and the cost-effectiveness plane for the comparison of the WinBUGS 2-state model with the 3-state model (using comparison of D + P with M + P). The CEACs showed that above approximately a £10,000 willingness-to-pay threshold, the probability that D + P was cost-effective was higher in the 3-state model than in the 2-state model. The cost-effectiveness plane showed that D + P was more effective (in terms of utility) than M + P for both the 2-state and 3-state models, although the uncertainty of its effectiveness was lower in the 3-state model than the 2-state model. However, the degree of uncertainty for the difference in cost of the 2 interventions was comparable for both models.

## Discussion

### Summary of Findings

We have investigated the use of multiparameter evidence synthesis, and in particular BRMA, for the purpose of synthesizing all relevant evidence to inform the health economic decision model. The methodology was applied to a motivating example of cost-effectiveness of docetaxel in combination with either prednisone or prednisolone (D + P) in mHRPC. At the time of the technology appraisal of docetaxel, only its effectiveness measured on OS but not PFS was reported, limiting the implementation of the Markov model to a 2-state model (alive and dead). We carried out research that investigated how inclusion of a predicted estimate of the effect of docetaxel on PFS would affect the cost-effectiveness estimates. We showed how such predicted estimate can be obtained from the BRMA and how it can enable implementation of a 3-state Markov model comprising stable disease state, separate progressive disease state, and dead state. By distinguishing alive patients between those who were stable and those who progressed, the model described the natural pathway of the disease in greater detail.

### Discussion of the Evidence Synthesis Methods

We used BRMA to predict the unreported treatment effect of docetaxel on PFS. This method assumes that the estimates of treatment effects on 2 outcomes, in our case log HR on OS and log HR on PFS, are correlated and follow a common bivariate normal distribution, as described by equation (1). These effects are estimates of true treatment effects in the population, which in the random-effects model for bivariate meta-analysis are also correlated and follow common bivariate normal distributions. The assumption of normality in the bivariate model is equivalent to assuming a linear relationship between the treatment effects on these 2 outcomes. Bivariate random-effects meta-analysis in the normal form has the advantage of borrowing strength between the treatment effects on the 2 outcomes, provided that the assumption of normality is reasonable. However, when such assumption cannot be made, using bivariate normal distribution can lead to “overshrinkage” and potentially to biased results.^33^ An alternative approach that can be used is a model developed by Daniels and Hughes^34^ for surrogate end point evaluation. This model assumes a bivariate normal distribution for the estimates of the treatment effect on the 2 outcomes and a linear relationship between the true treatment effects in the population. In contrast to the bivariate random-effects meta-analysis model, it does not make the assumption of the common distribution for the treatment effects on the first outcome (which is used to make the prediction from, in our case, the log HR on OS). Instead, it assumes fixed treatment effects on this outcome by placing separate prior distributions on these effects in each study, and a common normal distribution is only assumed for the second outcome. This relaxes the assumption of normality made in the random-effects but limits the borrowing of strength. Another approach that allows relaxing the exchangeability assumption is to use the *t* distribution (with low degrees of freedom) for the random-effects instead of the bivariate normal distribution in the bivariate meta-analysis,^9,35^ which can help when overshrinkage occurs. We discourage the use of simple meta-regression. Unlike the bivariate meta-analysis, it assumes that the treatment effect on the first outcome (in the case of our analysis, HR on OS) is measured without error (treats it as a fixed covariate) while in fact there is uncertainty associated with this effect related to the sample size of the RCT.^7^ This can lead to underestimating the uncertainty of the predicted treatment effect on PFS and, in consequence, the uncertainty of the cost-effectiveness estimates.

There were some limitations of our research. One was the variability in the definitions of the progression endpoints (for details, see Suppl. Appendix A.1). Standardizing the definition of the progression endpoint for all the RCTs, however, would require IPD from each of the trials, which is not achievable within the resources of this project.

Another limitation was a small number of studies in the BRMA. However, careful analysis of the results indicated that the predicted effect on PFS in TAX 327 had converged in the MCMC simulation. To further evaluate the validity of the method, we have carried out an analysis considering a scenario where the TAX 327 trial was replaced by the SWOG trial, for which treatment effects on both OS and PFS were reported. We conducted the analysis using data without the estimate of the treatment effect on PFS, and we predicted this effect in the same manner as we did for the TAX trial. This time, we were able to compare the results with the treatment effect reported by this trial. A full set of results is presented in Supplemental Appendix C.4. Although the treatment effect on PFS was overestimated (predicted HR, 0.623; 95% CrI, 0.397–0.927) in terms of the point estimate compared to the observed estimate (HR, 0.73; 95% CI, 0.627–0.860), the credible interval included the full range of values of the CI of the observed effect. Further investigation, reported in detail in Supplemental Appendix C.4, showed that one of the studies in the meta-analysis was an outlier (only with respect to PFS but not OS and therefore this did not affect any other results). Removing this study led to predicted HR PFS of 0.70 (0.46–1.04). The validation using the SWOG trial gave a similar result, which appears not very biased when compared to the observed estimate (see Suppl. Table SC7).

### Discussion of the Cost-effectiveness Modeling

The cost-effectiveness analysis resulted in a much lower ICER obtained from the 3-state model compared to the 2-state model. This was due to combining stable and progressed patients into a single state in the 2-state model, which likely underestimated the average utility. The 2-state model used utility reported by Sandblom et al.,^23^ who measured the EQ-5D 12 months prior to death. As discussed earlier, this utility value corresponds primarily to patients who progressed. Hence, the utility of the alive state in the 2-state model seems underestimated because some of the patients in this state, for a considerable amount of time, were progression-free and therefore should have higher utility. In the 3-state model, we used the same utility in the PD state as in the original 2-state model and allow for the utility in the StD state to vary over time, allowing a proportion of patients remaining in the StD state in each cycle to have higher utility, leading to higher average utility.

In addition, most patients leaving the StD state in the 3-state model transition to progression, and the difference in the transition probabilities between the treatments is defined by the HR of PFS. This difference is higher than in the 2-state model, where the transition probabilities correspond to the transition between alive and dead states and differ according to HR for OS (which is closer to 1 compared to HR for PFS). Therefore, the difference between treatments in rates of patients leaving the StD state in the 3-state model is also higher than the difference in the rates of patients leaving the alive state in the 2-state model. This leads to a larger average difference in utility, which is relatively larger than the difference in average incremental cost, as substantial care costs are still required after progression relative to the cost of treatment before progression. This substantial increase in the QALYs gained led to the much smaller ICER obtained from the 3-state model compared to the 2-state model. However, use of the 3-state model increased the overall level of uncertainty in the decision problem, as evidenced by a higher population EVPI. This was mainly driven by the increased uncertainty in the HR for D + P v. M + P for PFS compared to that for OS (used in the 2-state model). Further elaboration of the decision problem via use of a 3-state model could therefore enable more refined prioritization of future research that would not be otherwise possible with a 2-state model and could be formally assessed by the expected value of partial perfect information (EVPPI).^36^

As discussed earlier, the predicted HR for PFS for trial TAX 327 was likely to be an overestimate. We carried out a sensitivity analysis in which we used the predicted effect obtained from the sensitivity analysis, removing an outlier from the data used in the BRMA. Using this HR of 0.7 (0.46–1.04) led to a smaller difference in QALY (0.18 [0.05]), as well as a smaller difference in cost (£5258 [4238.03]), which resulted in an ICER of £29,601. More details can be found in Supplemental Appendix C5.1. This result highlights the sensitivity of the final result to the parameters of the model and in particular the predicted HR.

We also investigated the impact of the distribution of utility among the patients. To do so, we carried out a sensitivity analysis by implementing a new 2-state model with the cost and utility calculated in a more detailed way, similar to the 3-state model, allowing for some differences between stable and progressed patients to be taken into account. This led to a reduced ICER from £30,026 to £27,401. The details of the methods used and the results obtained from this sensitivity analysis are included in Supplemental Appendix C5.2. This method, however, did not model the natural pathway of the disease but only redistributed the data on utility in a more detailed way.

### Final Remarks and Conclusions

Ideally, when conducting an analysis for HTA decision making, analysts can contact the trialists to obtain the unreported estimates of effectiveness, which are needed to populate a decision model. However, this is not always possible. In the case of trial TAX 327, PFS was not set out to be reported in the trial. Occasionally, the treatment effect may not be reported due to outcome reporting bias or for other reasons, and it is not always possible to obtain estimates from the investigators, as found in the review of RCTs in NSCLC^2^ (see also the introduction). There are a number of initiatives under way to make data from clinical trials more accessible.^37^ Meanwhile, if obtaining IPD (or summary statistics) is not possible, our proposed method can serve to obtain relevant unreported estimates.

In conclusion, we have illustrated that a careful synthesis of all relevant evidence can allow valuable data on all outcomes, otherwise discarded, to be used to better inform the decision-making process. In the case study described here, although none of the studies included in the meta-analysis reported PFS for docetaxel, other studies investigating treatment effects of other treatments did report PFS, but these valuable data were not used to inform the health economic model. We would like to stress that our goal was not to critique the original approach to decision making by Collins et al.^12^ or to argue any particular result to be superior. In fact, there are many sources of uncertainty in the decision problem presented here. We would recommend that at least a sensitivity analysis to the structural assumptions of the model should be carried out, as recommended by Roberts et al.,^38^ particularly when data needed to populate the model are limited. We demonstrated that multivariate meta-analysis is a valuable tool in the synthesis of evidence for medical decision making that allows for inclusion of all or at least a wider range of available evidence on clinical effectiveness of interventions under assessment, potentially reducing the structural uncertainty of the decision model.

## Supplemental Material

DS_10.1177_0272989X18788537 – Supplemental material for Bayesian Multiparameter Evidence Synthesis to Inform Decision Making: A Case Study in Metastatic Hormone-Refractory Prostate Cancer Click here for additional data file. Supplemental material, DS_10.1177_0272989X18788537 for Bayesian Multiparameter Evidence Synthesis to Inform Decision Making: A Case Study in Metastatic Hormone-Refractory Prostate Cancer by Sze Huey Tan, Keith R. Abrams and Sylwia Bujkiewicz in Medical Decision Making
