# Supplementary material for: Bayesian Multiparameter Evidence Synthesis to Inform Decision Making: A Case Study in Metastatic Hormone-Refractory Prostate Cancer
Source: Med Decis Making. 2018 Aug 13;38(7):834–48. doi: 10.1177/0272989X18788537 (PMC6156771; doi:10.1177/0272989X18788537)
Supplement: DS_10.1177_0272989X18788537 – Supplemental material for Bayesian Multiparameter Evidence Synthesis to Inform Decision Making: A Case Study in Metastatic Hormone-Refractory Prostate Cancer [file DS_10.1177_0272989X18788537.pdf]

Supplementary Materials to

Bayesian multi-parameter evidence synthesis to inform decision-making - a case study in metastatic hormone-refractory prostate cancer

by Sze Huey Tan[1,2], PhD, Keith R. Abrams[1], PhD and Sylwia Bujkiewicz[1], PhD

[1]Biostatistics Research Group, Department of Health Sciences, University of Leicester, University Road, Leicester, LE1 7RH, UK

[2]Division of Clinical Trials and Epidemiological Sciences, National Cancer Centre Singapore, 11 Hospital Drive, Singapore 169610, Singapore

Appendix A: Summary of synthesis of evidence conducted by Collins et al

As part of the Health Technology Assessment (HTA) (Collins et al., 2007) evaluating the clinical- and cost-effectiveness of docetaxel in combination with either prednisone or prednisolone (D+P) for the treatment of metastatic hormone-refractory prostate cancer (mHRPC), a scoping search for studies evaluating the clinical- and cost-effectiveness of D+P was conducted. As only one RCT was identified to have compared D+P with mitoxantrone plus prednisone (M+P) and no other RCT compared D+P with any other possible interventions, RCTs that assessed mitoxantrone in combination with a corticosteroid compared with any chemotherapy regimen or best supportive care or placebo were also included in the scoping search. Extension of the studies selection to include studies that evaluated mitoxantrone in combination with a corticosteroid was to allow for the comparison between D+P and other relevant interventions using mitoxantrone in combination with a corticosteroid as a common comparator in indirect comparison analysis. In total, seven RCTs were identified based on the inclusion criteria, of which three RCTs used docetaxel compared with M+P, three RCTs used mitoxantrone plus a corticosteroid (M+P/H) compared with a corticosteroid (P/H) and one RCT used M+P compared with mitoxantrone plus prednisone plus clodronate (M+P+Clo). The three RCTs that included docetaxel had docetaxel in the following combination: D+P, docetaxel with estramustine (D+E) and docetaxel with estramustine and prednisone (D+E+P). All studies with details of interventions and reported outcomes are presented in Table A1.

Table A1: Studies included in the HTA report.

HTA Set	Trial	No. of arms	Reference Treatment	Comparative Treatment(s)	Total no. of patients	OS data	PFS data
Set 1*	CCI-NOV22 (Tannock et al., 1996)	2	M+P	P	161	Yes	Yes
	CALGB 9182 (Kantoff et al., 1999)	2	M+H	H	242	Yes	Yes
	Berry et al. (Berry et al., 2002)	2	M+P	P	120	Yes	Yes
	TAX 327 (Tannock et al., 2004)	3	M+P	D+P<br>D1+P	1006	Yes	No
Set 2†	Ernst (Ernst et al., 2003)	2	M+P	M+P+Cl	209	Yes	Yes
	SWOG (Petrylak et al., 2004)	2	M+P	D+E	674	Yes	Yes
	Oudard (Oudard et al., 2005)	3	M+P	D70+E+P<br>D35+E+P	127	Yes	Yes

*HTA set used in the main manuscript (licensed interventions)
†unlicensed interventions used by Collins et al. in sensitivity analysis (we use SWOG data to obtain some of the transition probabilities) .

A.1 Definitions of outcome measures

Definitions of OS were consistent for the four RCTs in HTA Set except for trial Berry, where OS was not explicitly defined (Berry et al., 2002). Overall survival was defined as the time from the date of randomisation to the date of death or censored at the date when the patient was last known to be alive. There were inconsistencies in the definition of progression across the three RCTs in HTA Set (excluding trial TAX327 which did not report PFS). Specifically, PFS reported for CALGB 918210 was defined as the time from the date of randomisation to the date of progression or death, whichever occurred first. Neither PFS nor time to progression (TTP) was reported for trial CCI-NOV2212; however, the HTA report presented TTP estimates for this trial. TTP was reported by Berry and colleagues (Berry et al., 2002) but no explicit definition for TTP was provided.

Appendix B: Cost-effectiveness analysis methods

For the cost-effectiveness assessment of D+P in the HTA report, two separate analyses were performed. This is due to the unlicensed status of some of the treatment regimens. For purpose of conducting the meta-analysis, Collins et al grouped both corticosteroids P and H together and we denoted them as P for presentation in the main manuscript and this supplement. The first analysis looked at three interventions (that are licensed at the time of the HTA report submission), namely D+P, M+P and P. The second analysis looked at eight interventions, including the three in the first analysis and the following five: D1+P, D+E, D70+E+P, D35+E+P, M+P+Clo. Due to the unlicensed status of the interventions in the second analysis, the economic decision making in the HTA report was based on the results of the first analysis. In the research that follows in this paper, the focus is on the cost-effectiveness assessment of the interventions compared in the first analysis. Data from SWOG trial were used to inform the transition probabilities in the cost-effectiveness analysis.

To develop the economic models, data for the construction of transition probabilities, definition of costs and utilities for each of the interventions need to be extracted. These data can be extracted from reviews, single RCT or evidence synthesis from a number of trials/studies. Specifications of the transition probabilities, cost and utilities are described in sections B.1, B.2 and B.3 respectively.

B.1 Transition probabilities

For the WinBUGS two-state model, the transition probabilities were estimated using the Weibull parameters reported in the HTA report, which used IPD from trial TAX 327. This was for consistency with the parameters used in the HTA model. As for the three-state model, which incorporated a PD state, the transition probabilities for transition from StD state to PD state were estimated using parametric Weibull survival modelling on reconstructed PFS IPD from one of the six RCTs (excluding trial TAX 327) in the HTA report. The selection criteria for the RCT to be used for estimating the transition probabilities for treatment arm M+P are: (i) comparable OS profile of the selected trial and trial TAX 327; (ii) selected trial having a mean time of progression closest to the reported mean cycle of M+P administrated in trial TAX 327 (that is: 5.9 cycles as reported in the HTA report). As PFS was not recorded for TAX 327, selection criteria (ii) is based on the assumption that the patients in trial TAX 327 M+P arm were administrated M+P till progression. The mean number of cycles of M+P administrated was then used as an approximation of the ‘potential’ mean time to progression for patients administrated M+P in trial TAX 327. Transition probability for transition from the StD state to dead state was obtained from an article on cost-effectiveness analysis for advance hormone-

dependent prostate cancer (Lu et al., 2012). In the absence of patient-level data on both OS and PFS for any of the interventions, the transition probability for transition from PD state to dead state could not be estimated. Methods for the estimation of (i) transition probabilities using parametric Weibull survival model and (ii) transition probabilities for transition from PD state to dead state in the three-state model are described in the next two subsections.

B.1.1 Transition probabilities estimated using parametric Weibull survival model

Survival analysis using the parametric Weibull model was used to implement time-dependency in the transition probabilities in the economic models (transition probabilities for transition from StD state to dead state in WinBUGS two-state model; and transition probabilities for transition from StD state to PD state in three-state model). The Weibull distribution takes the following probability density function:

$$f(t) = \lambda\gamma t^{\gamma-1} exp(-\lambda t\gamma)$$

where λ gives the scale of the distribution and γ defines the shape. The hazard function for this distribution is therefore:

$$h(t) = \lambda\gamma t^{\gamma-1}$$

with a cumulative hazard function of:

$$H(t) = \lambda t^{\gamma}$$

where the survival function is related to the cumulative hazard function in the following form:

$$S(t) = exp[-H(t)]$$

Since hazards are instantaneous, these need to be converted to a transition probability for a given period, such as a Markov cycle. Using the survival function, transition probability between time-points $(t-u)$ and t, denoted as $TP(t_u)$ where u is the cycle length, was defined as one minus the ratio of the survival function at the end of the interval to the survival function at the beginning of the interval. This function defined as:

$$TP(t_u) = 1 - S(t)/S(t-u)$$

was re-written using the cumulative hazed function as:

$$TP(t_u) = 1 - exp[-H(t)]/exp[-H(t-u)]$$

$$= 1 - exp[H(t-u) - H(t)]$$

Therefore, transition probability was defined using the Weibull parameters as follows:

$$TP(t_u) = 1 - exp[\lambda(t-u)^{\gamma} - \lambda t^{\gamma}]$$

In the HTA report, results of the Weibull survival analysis model were presented in the form of the regression coefficients of the intercept and scale parameters. These two parameters are expressed in terms of the Weibull parameters, λ and γ, as follows:

$$\lambda = exp(-\beta/\alpha)$$

$$\gamma = \frac{1}{\alpha}$$

where β is the intercept and α is the scale regression coefficient parameters from the Weibull survival analysis.

When performing the probabilistic analysis, the covariance between the intercept and scale regression parameter from the Weibull survival analysis were also incorporated in the WinBUGS two-state model. This was achieved by using the Cholesky decomposition matrix derived from the covariance matrix obtained from the Weibull survival regression model. Given a covariance matrix of the form:

$$Covariance, C = \begin{pmatrix} a & b \\ b & c \end{pmatrix}$$

the Cholesky decomposition matrix takes the form:

$$D = \begin{pmatrix} \sqrt{a} & 0 \\ \frac{b}{\sqrt{a}} & \sqrt{c - \frac{b^2}{a}} \end{pmatrix}$$

such that $C = D\,D^*$ where D^* denotes the conjugate transpose of D. Cholesky decomposition matrices of the covariance matrices for the interventions, D+P and M+P, were calculated independently and applied to the transition probabilities of D+P and M+P respectively in the WinBUGS two-state model to allow for the correlation between the intercept and scale parameters when sampling the random normal draws for the two parameters. Assuming that the Cholesky decomposition matrix of the covariance matrix for M+P is:

$$D_{M+P} = \begin{pmatrix} u_{D,M+P} & 0 \\ v_{D,M+P} & w_{D,M+P} \end{pmatrix}$$

the transition probability incorporating parameter uncertainties for transition from StD state to dead state for M+P is defined as:

$$TP_{D,M+P}(t_u) = 1 - exp[H_D(t-u) - H_D(t)]$$

$$TP_{D,M+P}(t_u) = 1 - exp\left[\lambda_{D,M+P}(t-u)^{\gamma_{D,M+P}} - \lambda_{D,M+P}t^{\gamma_{D,M+P}}\right]$$

where:

$$\lambda_{D,M+P} = \exp\left(\frac{-\beta_{D,M+P}}{\alpha_{D,M+P}}\right)$$

$$\gamma_{D,M+P} = \frac{1}{\alpha_{D,M+P}}$$

and

$$\beta_{D,M+P} = \beta_{M+P} + u_{D,M+P}Z_{\beta,D,M+P}$$

$$\alpha_{D,M+P} = \alpha_{M+P} + v_{D,M+P}Z_{\beta,D,M+P} + w_{D,M+P}Z_{\alpha,D,M+P}$$

where β_{M+P} and α_{M+P} are the intercept and scale regression coefficients for M+P presented in the HTA report; and $Z_{\beta,\mathrm{D},M+P} \sim Normal(0,1)$ and $Z_{\alpha,\mathrm{D},M+P} \sim Normal(0,1)$.

Transition probabilities of interventions D+P and M+P for the WinBUGS two-state model were calculated using the regression coefficients from the HTA report (Table 28 (Collins et al., 2007)). Transition probabilities for P were calculated by applying the HR of P versus M+P or HR of P versus D+P to the hazard rates of M+P and D+P in the transition probabilities respectively. Therefore, assuming that the transition probability for M+P is given by:

$$TP_{M+P}(t_u) = 1 - exp[H(t-u) - H(t)]$$

and with a HR for P versus M+P, denoted as $HR_{P/M+P}$, the transition probability for P is given by:

$$\begin{aligned} TP_P(t_u) &= 1 - exp\{HR_{P/M+P}[H(t-u) - H(t)]\} \\ &= 1 - exp[H(t-u) - H(t)]^{HR_{P/M+P}} \end{aligned}$$

Uncertainty associated with the HR was incorporated in the model by assigning a normal distribution to the logarithm of the HR as follows:

$$LHR \sim Normal(\bar{\mu}, \sigma^2)$$

where $\bar{\mu}$ and σ^2 are the mean and variance estimate of the log HR (LHR) from random-effects meta-analysis.

For the three-state model, the set of transition probabilities for intervention M+P was calculated using regression coefficients of the parameters of a Weibull survival model for PFS using re-constructed IPD from one of the RCTs in HTA Full Set selected based on the criteria outlined above. As no PFS patient-level data were available for the interventions D+P and P, transition probabilities for each of the interventions were calculated by applying their HR with respect to M+P to the transition probabilities of M+P. Similarly, uncertainty associated with each of the HRs was included in the respective models by assigning normal distribution to the LHRs.

B.1.2. Transition probabilities from PD state to dead state (three-state model)

Although IPD were reconstructed for PFS (together with OS) for the trial selected for estimating the transition probability from StD state to PD state, the reconstructed IPD for PFS and OS were not paired by patient. Hence, it would not be possible to estimate the transition probabilities from PD state to dead state using parametric survival analysis performed using reconstructed IPD as described in the previous section. To overcome this issue, transition probabilities were estimated by assuming the mean total survival time was equal to the weighted sum of combined survival time from stable disease to progression and then to death and the survival time when death occurred from other causes:

$$mean(Total\ Time) = W_{StDtoPDtoDead}[mean(Time_{StDtoPD}) + mean(Time_{PDtoDead})] + W_{StDtoDead}mean(Time_{StDtoDead})$$

where $mean(Time_{StDtoPD})$ defines the mean time that patients stayed in the StD state before transition to the PD state; $mean(Time_{PDtoDead})$ and $mean(Time_{StDtoDead})$ define the mean time for PD state to dead state and StD state to dead state respectively; W defines the weight assigned to the mean time and is related to the number of patients who transition through the two potential pathways in the economic model as shown in Figure 2 (bottom), from stable disease state to dead state either with or without disease progression.

As the proportion of patients who died of causes unrelated to prostate cancer was expected to be small (<1%), we assumed that $W_{StDtoDead} \rightarrow 0$, therefore $W_{StDtoPDtoDead} \rightarrow 1$. Hence,

$$mean(Total\ Time) = W_{StDtoPDtoDead}[mean(Time_{StDtoPD}) + mean(Time_{PDtoDead})]$$
$$= mean(Time_{StDtoPD}) + mean(Time_{PDtoDead})$$

and therefore,

$$mean(Time_{PDtoDead}) = mean(Total\ Time) - mean(Time_{StDtoPD})$$

Assuming that the survival rates for patients from PD to death follow an exponential survival distribution, the transition probability between time-points $(t-u)$ and t, denoted as $TP(t_u)$ where u is the cycle length, is defined as follows:

$$TP(t_u) = 1 - exp\{\lambda(t-u)^{\gamma} - \lambda t^{\gamma}\}$$
$$= 1 - exp(-\lambda u)$$

where $\gamma = 1$ for the exponential survival model.

As the hazard rate, $\lambda = \frac{1}{mean(Time)}$,

$$TP(t_u) = 1 - exp\left(\frac{-u}{mean(Time)}\right)$$

For M+P and D+P, the $mean(Total\ Time)$ for each of the interventions were estimated using the mean survival time calculated from the reconstructed OS IPD of trial TAX 327. For P, the mean survival time was estimated by a random-effect meta-analysis of the log hazard rate of the three RCTs that had a P treatment regimen arm.

As PFS endpoint was not recorded for trial TAX 327, the mean number of cycles of drug reported in the HTA report was used to represent the mean time from stable disease to progression, $mean(Time_{StDtoPD})$, based on the assumption that patients stopped drug treatment on the onset of disease progression. Mean number of cycles of drug P was not reported in the HTA report. Therefore, the mean time to progression was also estimated using meta-analysis of the log hazard rate of the two RCTs that reported PFS data for P.

Transition probabilities for transition from PD state to dead state for each intervention were therefore calculated using the equation:

$$TP(t_u) = 1 - exp\left(\frac{-u}{[mean(Total\ Time) - mean(Time_{StDtoPD})]}\right)$$

Uncertainty associated with the mean survival time or log hazard rate were also incorporated using normal distributions and propagated in the economic model. As the exponential survival

model is a single parameter model, Cholesky decomposition was not required for defining the uncertainty.

B.2 Cost

Cost data comprises drug acquisition and administration cost for each interventions, cost of the management of adverse side effects and subsequent follow up cost that included cost of further chemotherapy after disease progression, management of side-effects and palliative cost. Cost for each of the interventions to be used in the WinBUGS two-state and three-state model were extracted from cost data presented in the HTA report. In the report, costs were categorised into three components: namely, (i) the drug cost, (ii) the follow up cost and (iii) the terminal care cost. Drug cost included cost of acquisition and administration of each intervention.

Follow up cost included the cost of managing side-effects, subsequent chemotherapies and hospitalisation for palliative care. Terminal care costs were one-off costs used to incorporate the cost of caring for patients in the last month of life. As stated in the HTA report, terminal care cost data were not recorded in the trial (TAX 327), hence these costs were estimated from patients who died in the first six months after entering the trial. In the absence of costs per cycle for follow up cost, these costs were assigned and calculated as one-off cost, in a similar fashion as terminal care cost, as patient died. Cost data for interventions D+P and M+P were estimated using patient level data from trial TAX 327 while cost data for P were estimated using published cost-effectiveness analyses by Bloomfield and colleagues (Bloomfield et al., 1998).

Gamma distribution was used to represent uncertainty in the follow up costs and terminal care costs. Drug costs for each of the interventions were calculated based on the mean number of cycles of drugs administrated. Normal distribution was used to describe the number of cycles of drugs administrated to reflect uncertainty in the drug costs.

For the WinBUGS two-state model, the total costs were calculated as the summation of all three categories of costs. For the three-state model, drug costs and terminal care costs were calculated in a similar way to that calculated in the WinBUGS two-state model while follow-up costs were calculated by dividing the follow-up costs into two unequal parts (using a parameter ψ defined as in the equations that follow).

Costs of subsequent chemotherapy and hospitalisations accounted for between 70% and 80% of follow-up costs which most likely occurred post-progression and the remaining follow-up

cost (20% to 30%) were related to side effects likely to occur prior to progression (but may also be associated with the subsequent chemotherapy post-progression). Therefore the follow up costs were divided into portions corresponding to StD state and PD state.

As the base case analysis for the three-state model, 75% ($\psi = 0.75$) of the follow-up costs were assigned to the PD state to account for the cost of subsequent chemotherapy, managing side-effects and hospitalisations post-progression. Computation of these costs were based on the number of patients who died per cycle while the remaining 25% of the costs that were assigned to the StD state were computed based on the number of patients who progressed per cycle. Follow-up costs were assigned as one-off cost in a similar way as the WinBUGS two-state model.

In the WinBUGS two-state model, follow-up costs were calculated based on patients died per cycle as:

$$Total\ Cost_{FU} = \sum_{i=1}^{180} \left(Cost_{FU,i} \times N_{Died,i}\right)$$

where $Cost_{FU,i}$ represents follow-up cost data for cycle *i*, and $N_{Died,i}$ represents the number of patients who died in cycle *i*.

For the three-state model, the follow-up costs were calculated as:

$$Total\ Cost_{FU} = Total\ Cost_{StDFU} + Total\ Cost_{PDFU}$$

where:

$$Total\ Cost_{StDFU} = \sum_{i=1}^{180} \left[(1-\psi) \times Cost_{FU,i} \times N_{Progressed,i}\right]$$

$$Total\ Cost_{PDFU} = \sum_{i=1}^{180} \left(\psi \times Cost_{FU,i} \times N_{Died,i}\right)$$

ψ represents the proportion of follow-up costs associated with the PD state (termed “division factor”) and $N_{Progressed,i}$ represents the number of patients who progressed in cycle *i*.

An annual discount rate of 3.5% was used for discounting the cost after the first year.

B.3 Utility

EQ-5D values used in the cost-effectiveness analysis were obtained from Sandblom et al., who reported the EQ-5D values stratified according to whether the patients subsequently died due to cancer, due to other causes or were still alive, are listed in Table B1.

Utility for StD state in the WinBUGS two-state model was defined using a beta distribution with parameter values Beta(21.1, 18.1), derived from a mean EQ-5D HRQoL of 0.538 (95% CI: 0.461 to 0.615) as reported in the study by Sandblom and colleagues (Sandblom et al., 2004). For the three-state model, the utility distribution, Beta(21.1, 18.1), for the StD in the two-state model was assigned as the utility distribution for the PD state (denoted as U_{PD}) as the corresponding mean EQ-5D of 0.538 represents the HRQoL of all patients 12 months prior to death. The utility for the StD state was then calculated using EQ-5D values from Sandblom et al., (listed in Table B1 below) reported by subgroup according to whether the patients subsequently died due to cancer, due to other causes or were still alive. We used a weighted average of these values by splitting the patients in the StD state into three groups and using the following EQ-5D values from the Sandblom et al.: (i) EQ-5D values of all patients who died of other causes (denoted as $U_{Other\ causes}$) (ii) EQ-5D values of all patients who were still surviving with prostate cancer (denoted as $U_{Surviving}$) and lastly (iii) U_{PD} described earlier. These EQ-5D values, weighted by the transition probabilities amount to the utility for the StD state:

$$U_{StD} = TP_{StD}U_{Surviving} + TP_{StDtoPD}U_{PD} + TP_{StDtoDead}U_{Other\ causes}$$

where TP_{StD} is the probability of remaining in StD state, $TP_{StDtoPD}$ the probability of transition from StD state to PD state and $TP_{StDtoDead}$ the probability of transition from StD state to dead state without progression. A utility value of zero was assigned to the dead state ($U_{Dead} = 0$).

The value of the utility for the StD state was calculated using the utility for progressed patients (as stated above for the two-state model) and two additional EQ-5D values extracted from the Sandblom study (Sandblom et al., 2004), they were the EQ-5D for patients who were still surviving at the time of analysis of the study, EQ-5D = 0.770 (95% CI: 0.755 to 0.785), and EQ-5D of patients who died of other non-prostate cancer-related death, EQ-5D = 0.564 (95% CI: 0.497 to 0.631). These two utility data were defined in the economic model using the following beta distributions, Beta(581.3, 173.6) and Beta(29.1, 22.5) respectively to derive the utility for the StD state.

Table B1: Utility values, obtained from Table 1 in Sandblom et. al.

	Died of prostate cancer	Died of other causes	Still alive 31 December 2000
Eq5D score (± 95% CI)	0.538 ± 0.077	0.564 ± 0.067	0.770 ± 0.015

As the utilities defined were selected to reflect the general HRQoL of patients with advanced prostate cancer and were independent of the interventions administrated by the patients, the utilities were used in the model for all three interventions.

B.4 Cost effectiveness

Cost effectiveness of interventions was assessed by obtaining the incremental cost-effectiveness ratios (ICER). ICER was calculated by taking the difference between the mean values of the cost of interventions over the difference between the mean values of the QALYs gained of interventions as:

$$ICER = \frac{\overline{Cost_{D+P}} - \overline{Cost_{M+P}}}{\overline{QALY_{D+P}} - \overline{QALY_{M+P}}}$$

where $\overline{Cost_{D+P}}$ and $\overline{Cost_{M+P}}$ defines the mean cost of D+P and M+P respectively and $\overline{QALY_{D+P}}$ and $\overline{QALY_{M+P}}$ defines the mean QALY gained per patient for D+P and M+P respectively.

Appendix C: Additional results

C.1 Re-constructed IPD summary statistics

Tables C1 and C2 show HRs for OS and PFS respectively, for all seven studies listed in Table A1 for completeness.

Table C1: Individual trial's HRs on OS obtained using IPD reconstructed from Kaplan-Meier survival curves

Trial	Comparison	HR (95% CI) reported in journal article	HR (95% CI) reported in HTA report	HR (95% CrI) from reconstructed IPD
Overall Survival				
TAX 327	D+P / M+P	0.76 (0.62-0.94)	0.76 (0.62, 0.94)	0.76 (0.620, 0.936)
CALGB 9182	M+H / H	Not reported but median survival reported as: M+H 12.3 months and; H 12.6 months (p=0.77)	1.05 (0.74, 1.49)	0.96 (0.732, 1.251)
CCI-NOV 22	M+P / P	Not reported but a total of 140 deaths reported at time of analysis (p=0.27)	0.91 (0.69, 1.19)	0.81 (0.590, 1.110)
Berry	M+P / P	Not reported but median survival reported as: M+P 23 months and; P 19 months (p=0.569)	1.13 (0.75, 1.70)	0.95 (0.628, 1.432)
Ernst	M+P+Cl/M+P	1.05 (0.78, 1.42)	1.05 (0.78, 1.42)	1.08 (0.799, 1.452)
SWOG	D+E / M+P	0.8 (0.67, 0.97)	0.8 (0.67, 0.97)	0.79 (0.659, 0.955)
Oudard	D70+E+P / M+P	Not reported but median survival reported as: D70+E+P 18.6 months, D35+E+P 18.4 months and; M+P 13.4 months	0.94 (0.29, 1.02)	1.08 (0.675, 1.715)
	D35+E+P / M+P		0.86 (0.68, 1.08)	0.75 (0.448, 1.245)

Table C2: Individual trial's HRs on PFS obtained using IPD reconstructed from Kaplan-Meier survival curves

Trial	Comparison	HR (95% CI) reported in journal article	HR (95% CI) reported in HTA report	HR (95% CrI) from reconstructed IPD
Progression-free Survival				
TAX 327	D+P / M+P	Endpoint not collected	Not possible	Not possible
CALGB 9182	M+H / H	Not reported but median survival reported as: M+H 3.7 months and; H 2.3 months (p=0.0218)	Time to progression (calculated from number of events and p-value presented in the trial publication) HR= 1.50 (1.06, 2.13); p = 0.0218	0.74 (0.574, 0.954)
CCI-NOV 22*	M+P / P	Not reported	0.47 (0.32, 0.68)	Not possible+
Berry*	M+P / P	Not reported but median survival reported as: M+P 8.1 months and; P 4.1 months (p=0.018)	Estimated from the Kaplan-Meier curve for PFS presented in the trial publication. HR= 0.64 (0.48, 0.86)	0.63 (0.432, 0.927)
Ernst	M+P+Cl/M+P	0.81 (0.61, 1.07)	0.81 (0.61, 1.07)	0.84 (0.63, 1.112)
SWOG	D+E / M+P	Not reported but median survival reported as: D+E 6.3 months and; M+P 3.2 months (p<0.001)	time to disease progression observed for the docetaxel group compared with the mitoxantrone group: HR=1.30 (1.11, 1.52); p < 0.001	0.73 (0.627, 0.860)
Oudard*	D70+E+P / M+P<br>D735+E+P / M+P	Not reported but median survival for time to PSA progression is reported as: D70+E+P 8.8 months, D35+E+P 9.3 months and; M+P 1.7 months	Not reported<br>Not reported	Not possible<br>Not possible

*Trials where TTP was reported in the journal article or HTA report instead of PFS
+No Kaplan-Meier survival curve in published article

C.2 Justification for choice of Trial SWOG

Overall survival curves for four RCTs in HTA Set 1 and Set 2 (excluding CALGB 9182 which used hydrocortisone instead of prednisone and CCI-NOV22 which did not report PFS) were compared to the OS curve of trial TAX 327 and are presented in Figure C1. The OS Kaplan-Meier curves suggested that trial SWOG has an OS profile closest to trial TAX 327.

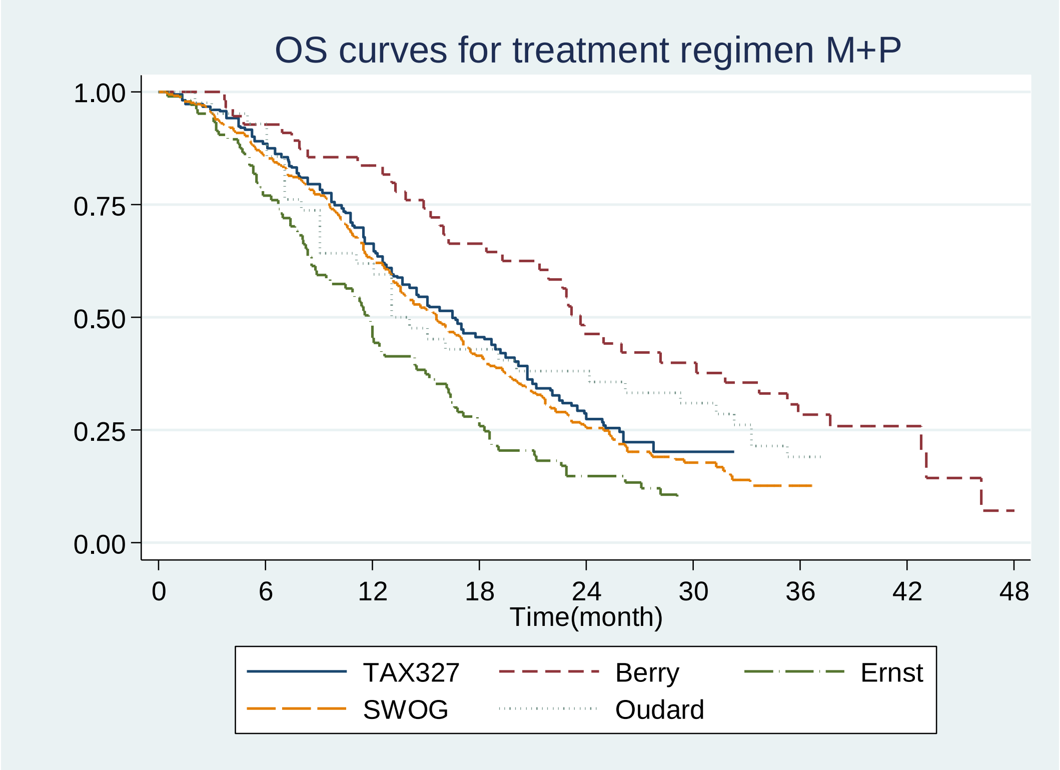

Figure C1: Overall survival Kaplan-Meier curves for RCTs in the HTA report

Mean time to progression for patients administrated M+P for the four RCTs were estimated using the IPD reconstructed from PFS Kaplan-Meier curves. As PFS was not recorded in trial TAX 327, mean number of cycles of M+P administrated in trial TAX 327 was used for comparing with the mean time to progression in the four RCTs. The mean time to progression for the four RCTs and the mean number of cycles of M+P administrated in trial TAX 327 are shown in Table C3. Trials Ernst and SWOG have mean time to progression closest to the assumed mean time to progression of TAX 327. The results suggested that trial SWOG has an OS profile closest to trial TAX 327 and therefore potentially a PFS profile closest to TAX 327 if the PFS endpoint had been recorded. Hence, reconstructed IPD of trial SWOG were used to estimate the transition probabilities from StD state to PD state for M+P.

Table C3: Mean time to progression for RCTs in the HTA report

Trial	Mean Time to Progression (SE)
Berry et al.	12.8 (1.63)
Ernst	5.9 (0.53)
SWOG	5.9 (0.33)
Oudard	4.2 (0.88)
	Mean no.of cycles (SE)
TAX 327	5.9 (0.17)

C.3 Cost and utility

Costs of drug for interventions M+P and D+P were estimated using the mean cycle of chemotherapy administrated. The reported mean number of cycles for the interventions M+P and D+P were 5.9 (SE=0.17) and 7.3 (SE=0.18) respectively, Normal distributions were assigned to the number of treatment cycles to incorporate uncertainty around these values in the two economic models. Cost of drug for M+P was £347.73/cycle and £1253.92/cycle for D+P, including £177.46/cycle for outpatient attendance fees for both drug regimens. Cost of drug for P was calculated at £1.48 per patient per cycle. In the WinBUGS two-state model, the drug costs for a patient taking P was calculated for the number of cycles that the patient remains in the StD state before transition to the dead state. In the three-state model, the cost drug for a patient taking P was calculated for the number of cycles that the patient remains in the StD state before transition to the PD state. It was assumed that the patient would stop taking P after progression and hence, no drug cost would be calculated for the cycles post-progression before transition to the dead state.

Follow-up and terminal care costs for interventions M+P and D+P were estimated from trial TAX 327 as reported in the HTA report (Table 36 and Table 37 in (Collins et al., 2007)). Uncertainties for the costs were applied in the two economic models using Gamma distributions. Follow-up and terminal care costs for drug P were not available and were estimated from the costs of intervention M+P from trial TAX 327. In order to estimate the costs for drug P, a cost ratio of drug P with reference to drug M+P was estimated using costing data of P and M+P from a review article (Bloomfield et al., 1998). The mean cost ratio estimated in the WinBUGS two-state model was 1.278 (95% CrI: 0.946 to 1.691) which suggested that the mean cost of P was higher than the mean cost of M+P. This cost ratio was calculated by assigning Gamma distributions [Gamma(α,β)] of Gamma(105, 276) and Gamma(81, 285) to the cost data of P and M+P respectively. The mean cost (drug, follow-up and terminal care)

per patient at each state in the economic model for each of the interventions are presented in Table C4.

Mean EQ-5D HRQoL for all patients in the 12 months prior to death was 0.538 (95% CI: 0.461 to 0.615) as reported in the study by Sandblom and colleagues (Sandblom et al., 2004). Using this EQ-5D data, the utility for StD state in the WinBUGS two-state model was defined using a beta distribution with parameter values: Beta(21.1, 18.1). For the three-state model, the utility distribution, Beta(21.1, 18.1), for the StD in the WinBUGS two-state model was assigned as the utility distribution for the PD state. Two additional EQ-5D values as discussed in Section 2.3.3 of the main manuscript were extracted from the Sandblom study (Sandblom et al., 2004), they were the EQ-5D for patients who were still surviving at the time of analysis of the study, EQ-5D = 0.770 (95% CI: 0.755 to 0.785), and EQ-5D of patients who died of other non-prostate cancer-related death, EQ-5D = 0.564 (95% CI: 0.497 to 0.631). These two utility data were defined in the economic model using the following beta distributions, Beta(581.3, 173.6) and Beta(29.1, 22.5) respectively. Utility for the StD state was calculated based on the method described in Section 2.3.3 of the main manuscript.

As the utilities defined were selected to reflect the general HRQoL of patients with advanced prostate cancer and were independent of the interventions administrated by the patients, the utilities were used in the model for all three interventions. Mean QALY per patient for each of the interventions in the economic models are presented in Table C4.

Table C4: Mean cost, mean QALY and mean time spent per patient at each state in the economic model

Economic Model	Drug	Mean Cost (£) (95% CrI)	Drug cost (£)	Mean QALYs (95% CrI)	Mean Time (95% CrI)
WinBUGS two-state model					
Alive State	P (direct)	11772 (6127, 20280)	26 (23, 31)	0.809 (0.5590, 1.0760)	18.1 (15.54, 21.03)
	M+P	11237 (6855, 17030)	2057 (427, 3679)	0.813 (0.5718, 1.0580)	18.2 (16.55, 19.93)
	D+P	15862 (9066, 23020)	9152 (3261, 15050)	0.967 (0.6746, 1.2690)	21.9 (19.50, 24.58)
WinBUGS three-state model					
Stable Disease State	P (direct)	NA	6 (1, 18)	0.276 (0.0614, 0.7646)	4.1 (0.68, 12.03)
Progression Disease State	P (direct)	NA	mean cycles = 4.12 (SE:1.47)	0.573 (0.2667, 1.0080)	13.4 (6.63, 22.81)
Total (for P)		10152 (5160.0, 17760.0)#		0.849 (0.4389, 1.4400)	17.5 (9.32, 28.90)
Stable Disease State	M+P	371 (211.6, 589.9)	2047 (400, 3678)	0.377 (0.3330, 0.4247)	5.7 (5.05, 6.40)
Progression Disease State	M+P	3804 (2088.0, 6345.0)	mean cycles = 5.34 (SE:0.17)	0.512 (0.3596, 0.6674)	11.9 (10.78, 13.12)
Terminal care	M+P	3756 (1026.0, 8239.0)			
Total (for M+P)		9977 (5995.0, 15250.0)		0.889 (0.7200, 1.0600)	17.6 (16.32, 18.99)
Stable Disease State	D+P	367 (218.6, 562.7)	9164 (3268, 15000)	0.608 (0.5097, 0.7196)	9.5 (7.89, 11.24)
Progression Disease State	D+P	2467 (1368.0, 3991.0)	mean cycles = 6.62 (SE:0.26)	0.522 (0.3669, 0.6803)	12.4 (11.17, 13.56)
Terminal care	D+P	3327 (916.5, 7268.0)			
Total (for D+P)		15327 (8589.0, 22420.0)		1.131 (0.9391, 1.3260)	21.8 (19.90, 23.86)

#Calculated using mean cost ratio of 1.278 (95% CrI: 0.946 to 1.691) for P compared to M+P

C.4 Validity of the use of BRMA to predict treatment effect of docetaxel on PFS

Table C5 shows the results of the case study where the treatment effect on PFS (hazard ratio and the corresponding 95% CrI) was predicted from the treatment effect on OS, conditional on the data for the treatment effects on both outcomes in the three studies in the HTA set (Table A1) reporting both outcomes. The predicted effects on PFS are obtained for TAX 327, used in the main manuscript and for SWOG trial. The SWOG trial was not part of the main analysis as it included an unlicensed intervention. However, as discussed in section C2, the trial was similar to TAX 327 and it reported the treatment effect on PFS. Therefore it was possible to compare the predicted effect on PFS for the SWOG trial (obtained from the BRMA without using the available PFS information) to the actual trial result (generated according to available PFS information).

Table C5: Effectiveness estimates for TAX 327 and SWOG trials including the predicted HR on PFS, obtained from the BRMA

Trial	Re-constructed HR OS	Re-constructed HR PFS	Predicted HR PFS
TAX327	0.76 (0.620,0.936)	NA	0.619 (0.393, 0.924)
SWOG	0.79 (0.659,0.955)	0.73 (0.627,0.860)	0.623 (0.397, 0.927)

The predicted effect on PFS for the SWOG trial (HR=0.623 (95% CrI: 0.397 – 0.927) was overestimated in comparison with the actual results: HR=0.73 95% CI: (0.627 – 0.860). However, the predicted interval included the full range of values of the CI of the actual estimate of the treatment effect on PFS. The predicted effect was obtained with higher uncertainty.

When comparing the predicted treatment effect on PFS for the TAX 327 trial to the predicted effect for the SWOG trial, the results are similar. The HR (PFS) for the TAX 327 trial (HR=0.619) is minimally larger than the predicted effect for the SWOG trial (HR=0.623), which is proportional to the difference in the treatment effects on OS between the two trials (for OS: HR=0.76 for TAX 327 and HR=0.79 for SWOG trial).

We conducted further investigation by including all trials from both sets: HTA Set1 and HTA Set2 (see Table A1) which reported treatment effects on both PFS and OS. This resulted in a new data set of five trials: CALGB 9182, CCI-NOV 22, Berry, SWOG and Ernst. We carried out a cross-validation procedure, taking in one study at a time the treatment effect on PFS (assuming not reported) and predicting this effect using the bivariate meta-analysis of the remaining data on the two outcomes. Results are included in Table C6. The predicted values in all studies were similar apart from study CCI-NOV 22. Investigating the results in Table C6

made it apparent that study CCI-NOV 22 was an outlier (the smallest HR for PFS) generating bias when making the predictions.

Table C6: Predicted HR for PFS obtained in a cross-validation procedure

Trial which predicted PFS corresponds to	Re-constructed HR for OS	Re-constructed HR for PFS	Predicted HR for PFS
CALGB 9182	0.96 (0.72, 1.25)	0.74 (0.57, 0.95)	0.68 (0.45, 1.02)
CCI-NOV 22	0.81 (0.59, 1.11)	0.47 (0.32, 0.68)^	0.73 (0.51, 1.03)
Berry	0.95 (0.63, 1.43)	0.63 (0.43, 0.93)	0.70 (0.45, 1.09)
SWOG	0.79 (0.66,0.96)	0.73 (0.63,0.86)	0.67 (0.42, 0.98)
Ernst	1.08 (0.80,1.45)	0.84 (0.63,1.11)	0.67 (0.43, 1.01)

^Kaplan-Meier curve for PFS not available, HR shown was extracted from HTA report.

Following this we carried out a sensitivity analysis removing the study CCI-NOV 22 from the main analysis used to predict HR on PFS for TAX 327 trial. The predicted value of HR for PFS for TAX 327 along with the prediction for SWOG trial used for validation are shown in Table C7.

Table C7: Effectiveness estimates for TAX 327 and SWOG trials including the predicted HR on PFS, obtained from the bivariate meta-analysis of three trial: TAX 327 (or SWOG) with CALGB 9182 and Berry

Trial	Re-constructed HR for OS	Re-constructed HR for PFS	Predicted HR PFS
TAX327	0.76 (0.620,0.936)	NA	0.698 (0.458, 1.036)
SWOG	0.79 (0.659,0.955)	0.73 (0.627,0.860)	0.695 (0.468, 1.017)

C.5 Sensitivity analyses for the cost-effectiveness modelling

C.5.1 Sensitivity analysis when using alternative predicted values for HR for PFS

We carried out three sets of sensitivity analysis using three sets of data when predicting HR for PFS in TAX 327. First we increased the evidence base containing the three HTA studies (denoted as “3 HTA” in Table C8) reporting both outcomes and the TAX 327 trial by adding SWOG trial to the data set. This was done to reduce the impact of the outlier (see section C.4). We then increased evidence base further by including Ernst study (SWOG and Ernst studies were the two studies in HTA Set2 reporting both outcomes). Both analyses resulted in

reduced treatment effect difference on PFS (predicted HR closer to 1.00) and higher ICERs of £26,483 and £28,349 respectively. We then carried out the sensitivity analysis using reduced evidence base by removing the outlier from the data set in the main analysis (as in Table C7, denoted in Table C8 as “2 HTA”). This led to a higher ICER of £29,601. All results are shown in Table C8 with the original results (on the left-hand-side) listed for comparison.

Table C8: Sensitivity analysis for the three-state model

Data source	3 HTA+ TAX327	3 HTA+ TAX327+SWOG	3 HTA+ TAX327+ SWOG + Ernst	2 HTA+ TAX327 (Ex. CCI-NOV22)
Predicted HR for PFS	0.62 (0.39, 0.92)	0.67(0.45, 0.93)	0.69(0.48, 0.93)	0.70 (0.46, 1.04)
Difference in Cost, Mean (SE)	£5,349 (4243.53)	£5,291 (4239.96)	£5,270 (4238.78)	£5,258 (4238.03)
Difference in QALY, Mean (SE)	0.242 (0.0526)	0.200 (0.0503)	0.186 (0.0496)	0.178 (0.0474)
ICER	£22,148	£26,483	£28,349	£29,601

Differences were calculated for (D+P) versus (M+P)

<u>*C.5.2 Sensitivity analysis for 2-state model with alternative approach to utility and cost*</u>

We have carried out a sensitivity analysis to illustrate how the results of the economic model will change when the 2-state model is implemented, but the utility and cost is calculated to account for the differences between those patients who progressed and those who did not progress. To obtain the utility for the Alive state in the two state model, we use the values from Sandblom in Table B2 corresponding to patient who died due to prostate cancer and those who remained alive at the end of the Sandblom study averaged with weights corresponding to the transition probabilities for the transition out of the Alive state:

$$U_{Alive} = TP_{Alive} U_{Surviving} + TP_{AlivetoDead} U_{PD},$$

similarly as in the three state model. The utility of patient who died of other causes is not included here separately, because in the two-state model transition probability to dead state corresponds to the all-cause mortality and $U_{Other\ causes}$ had almost the same value as U_{PD}. Therefore U_{PD} includes both set of patients.

In a similar manner we partition the cost of each treatment assigned to the StD state. Recalling from section B.2, between 70% and 80% of the costs of follow-up was assumed to be related to subsequent chemotherapy and hospitalisations (likely occurring post-progression) and the

remaining follow-up cost (20% to 30%) were related to side effects (likely occurring prior to progression). Therefore in the three state model, the follow up costs were divided into portions corresponding to StD state and PD state. In a similar manner to divide this cost in the new two-state model, averaging the portions of the cost by the transition probabilities:

$$C_{Alive} = TP_{Alive}C_{Surviving} + TP_{AlivetoDead}C_{PD}$$

where $C_{Surviving} = 0.25C_{Follow\ up}$ and $C_{PD} = 0.75C_{Follow\ up}$.

Results from this alternative two-state model are included in Table C9 (middle column) along with the results from the original two-state model and the three-state model for comparison.

Table C9: Sensitivity analysis of the new two-state model (middle column), along with the original two-state model (left-hand-side results) and three-state model (right-hand-side column) for comparison

	WinBUGS two-state model	Sensitivity Analysis New two-state model (Utility+Cost)	WinBUGS three-state model
Difference in Cost	£4,624 (4407.83)	£6,038 (4032.68)	£5,349 (4243.53)
Difference in QALY	0.154 (0.0676)	0.220 (0.0902)	0.242 (0.0526)
ICER	£30,026	£27,401	£22,148
